# Basal Insulin Reduces Glucose Variability and Hypoglycaemia Compared to Premixed Insulin in Type 2 Diabetes Patients: A Study Based on Continuous Glucose Monitoring Systems

**DOI:** 10.3389/fendo.2022.791439

**Published:** 2022-04-27

**Authors:** Huiying Wang, Yunting Zhou, Yuming Wang, Tingting Cai, Yun Hu, Ting Jing, Bo Ding, Xiaofei Su, Huiqin Li, Jianhua Ma

**Affiliations:** Department of Endocrinology, Nanjing First Hospital, Nanjing Medical University, Nanjing, China

**Keywords:** basal insulin, premixed insulin, continuous glucose monitoring, glycaemic variability, T2DM, hypoglycaemia

## Abstract

**Aims:**

To examine the glycaemic variability and safety of basal and premixed insulin by using continuous glucose monitoring (CGM) systems.

**Methods:**

393 patients with type 2 diabetes mellitus (T2DM) treated with basal or premixed insulin for more than 3 months were enrolled. Patients were classified into a basal insulin group or premixed insulin group according to their insulin regimens. CGMs were used for 72 h with their previous hypoglycaemic regimen unchanged. The following glycaemic parameters were calculated for each 24 h using CGM data.

**Results:**

Despite similar HbA1c and fasting C-peptide concentrations, glycaemic variability (GV), including the mean amplitude of glycaemic excursion (MAGE), standard deviation (SD) and coefficient of variation (CV), and the time below range (TBR) were significantly lower in the basal insulin group than these in the premixed insulin group. Night-time hypoglycaemia was lower in the basal insulin group than that in the premixed insulin group (p<0.01). Among participants with haemoglobin A1c (HbA1c) < 7%, the GV and TBR were higher in the premixed insulin group than that in the basal insulin group.

**Conclusion:**

Compared with basal insulin, the patients who use premixed insulin had higher GV, smaller TIR and an increased incidence of hypoglycaemia. For patients who use premixed insulin and with HbA1c < 7%, more attention needs to be given to hypoglycaemic events and asymptomatic hypoglycaemia.

**Clinical Trial Registration:**

ClinicalTrials.gov, identifier NCT03566472.

## Introduction

Type 2 diabetes mellitus (T2DM), characterized by insulin resistance and insulin secretion deficiency (1), is rising at an alarming rate. The prevalence of diabetes in China has increased from 0.67% in 1980 to 12.8% in 2017 (2). In China, all diabetes care is provided by hospital specialists. The current treatment paradigm of T2DM is gradual regimens intensification (3). When lifestyle modification and oral antidiabetic drugs fail to achieve adequate glycaemic control, many patients eventually require and benefit from insulin therapy (4).

Guidelines recommend that insulin therapy should be initiated timely in patients with a long duration of diabetes, use of oral hypoglycaemic drugs that fail to achieve goals and poor islet function. It is known that Chinese diets are carbohydrate-heavy, and β-cell function is generally poorer in Chinese individuals (5). Depending on the patient’s illness and the physician’s practice, premixed insulin therapy and basal insulin therapy are both recommended for initiation for initial insulin therapy to maintain their blood glucose concentrations in the target range in China (6, 7). Due to its lower price than basal insulin, premixed insulins are more widely used as the starting insulin therapy in clinical practice (8, 9).

In an attempt to reach glycaemic targets, patients who are treated with premixed insulin usually require an increased number of injections. Unfortunately, researchers found that a high proportion of patients still did not achieve the goal that HbA1c levels are lower than 7%, and this treatment regimen might be associated with a higher risk of hypoglycaemic episodes and weight gain (10). A randomized clinical trial reported that T2DM patients who were treated with premixed insulin had glycaemic control similar to that of patients treated with a basal insulin regimen but had a significantly higher frequency of hypoglycaemia (11).

Continuous glucose monitoring (CGM) systems have been recognized as an ideal method of monitoring glycaemic control in diabetic patients (12). The data of rigorous 24 h glucose profiles from CGM allowed the calculation of glycaemic variations, detection of asymptomatic hypoglycaemia (Without typical symptoms of hypoglycaemia but plasma glucose measurements ≤ 3.9 mmol/L) and accurately depict the characteristics of blood glucose fluctuations (13). At present, few studies have reported the use of CGM to observe the effects of basal insulin and premixed insulin on the glycaemic profile in T2DM patients. Thus, this study was conducted to investigate the differences in glycaemic variability and hypoglycaemia between basal insulin and premixed insulin by using CGM.

## Patients and Methods

### Participants

In this cross-sectional observation study, 393 outpatients with T2DM who had been treated with basal insulin or premixed insulin were enrolled at Nanjing First Hospital from July 2019 to December 2020.

The inclusion criteria were as follows (1): patients diagnosed with T2DM as defined by the World Health Organization in 1999; (2) patients aged ≥ 18 years; (3) body mass index (BMI) between 19 and 35 kg/m^2^; (4) patients using basal insulin or premixed insulin (daily dose > 0.2 IU/kg/day) for more than 3 months; (5) patients with relatively consistent diet and exercise habits during the study period.

The following patients were excluded: (1) patients with type 1 diabetes mellitus; (2) patients with serious acute and/or chronic complications, including ketoacidosis, hyperosmolar state, end-stage renal disease, and severe cardiovascular diseases; (3) patients with severe infectious diseases; (4) patients with known cancers; (5) patients with cognitive disorders, drug abuse, or alcoholism.

### Study Design

**Figure 1:**
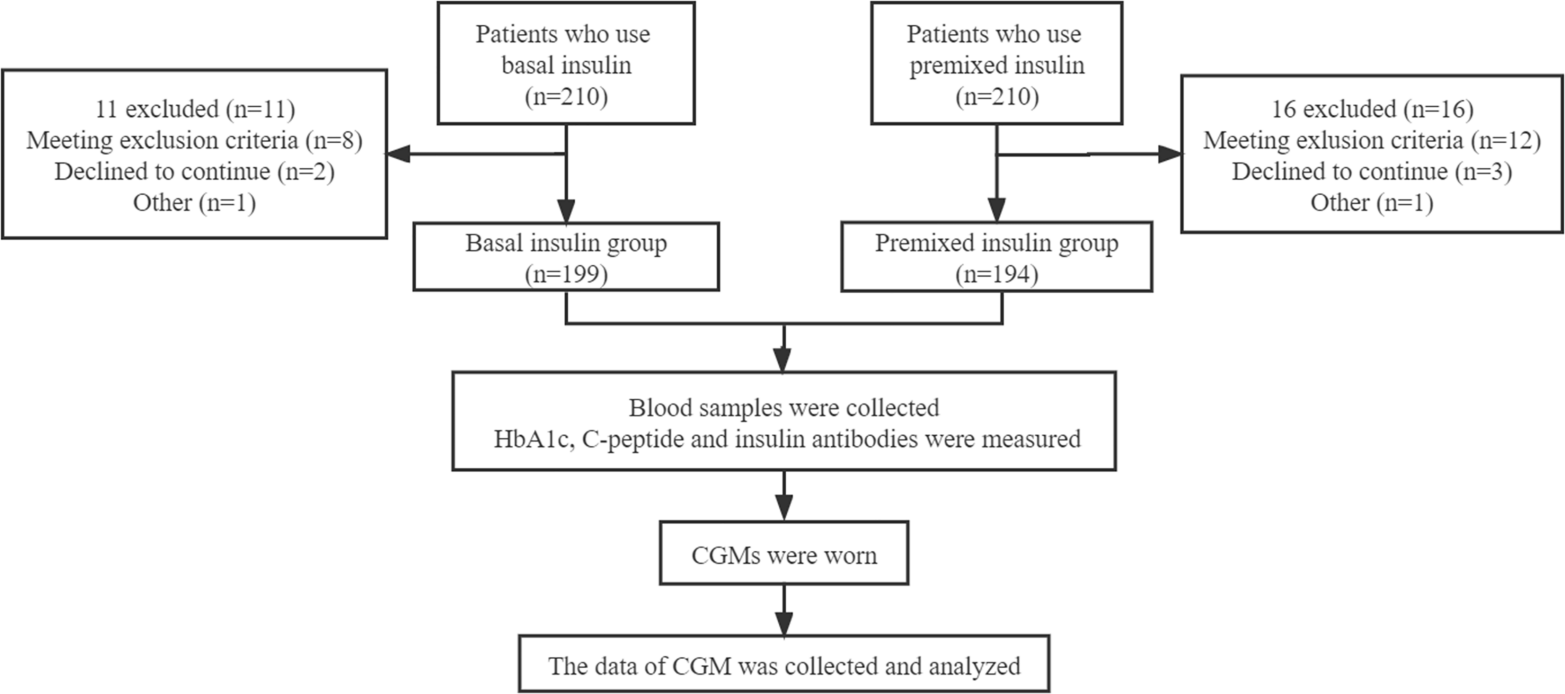
Flow chart.

Written informed consent has signed by each participant. The study protocol was conducted in accordance with the 1964 Helsinki Declaration and its later amendments or comparable ethical standards. General information (such as age, sex, duration of T2DM, types and dosage of oral hypoglycaemic medication, insulin type and insulin dose) of the patients was collected by trained doctors (**Figure 1**).

393 patients who used basal insulin or premixed insulin for more than 3 months were classified into the basal insulin group (199 cases) or premixed insulin group (194 cases) according to their insulin regimen. The basal insulin used in this study is insulin glargine, including Basalin and Lantus, both of which are insulin analogues. Insulin types of premixed insulin group were Mixed Protamine Zinc Recombinant Human Insulin Injection, Biosynthetic Human Insulin Injection, Insulin Aspart 30 Injection, Mixed Protamine Zinc Recombinant Human Insulin Lispro Injection (50R), Mixed Protamine Zinc Recombinant Human Insulin Lispro Injection (25R) respectively. Blood samples from all patients were collected after fasting more than 10 h overnight. HbA1c was measured using a high-performance liquid chromatography assay (Bio-Rad Laboratories, Inc. CA, USA), C-peptide was assessed by ECLIA immunoassay analyzer Elecsys170 (Roche, Germany). Each sample for insulin antibodies measurement was run in duplicate, and optical density (OD) by ECLIA semi-quantitative assay. An index identified as COI was calculated based on the average of the results of each sample for Ins-Ab: COI = OD/CO (OD of test sample)/(OD of average absorbance of negative control) using a previously described method with some modification (14). The reference range of normal values for antibody is <1 COI.

The CGM in this study were retrospective CGM (Medtronic Mini Med), which were worn for 72 h. All patients were educated and provided with the CGM device by endocrine specialist nurses. Glucose values of peripheral blood were entered into CGM to calibrate device four times a day. The data of CGM was blinded to all the subjects. The patients maintained consistent diet and exercise habits, and recorded any incidences of hypoglycaemia (blood glucose level < 3.9 mmol/L), allergic reactions, and other abnormalities during the study period. Patients were advised to eat if they experienced asymptomatic hypoglycaemia or symptomatic hypoglycaemia (Typical symptoms of hypoglycaemia with plasma glucose concentration ≤ 3.9 mmol/L). If severe hypoglycaemia (Require help from others to administer glucose, inject glucagon, or take any other corrective action) occurred, the researchers would adjust the insulin dose of the participants.

To improve statistical power, we combined all patients and divided all patients (both the basal insulin group and premixed insulin group) into three groups according to the level of HbA1c, namely as HbA1c < 7%, 7% ≤ HbA1c ≤ 9%, HbA1c > 9%. At last, we sub-divided patients according to HbA1c within the basal insulin group (HbA1c < 7% and HbA1c ≥ 7%) and premixed insulin group (HbA1c < 7% and HbA1c ≥ 7%).

### CGM

Data was collected from 00:00 to 24:00 on the second day of CGM. The following parameters were calculated: 1) 24-h mean blood glucose (MBG); 2) the mean fluctuation amplitude value from peak to valley every 24 h (24-h MAGE); 3) Standard deviation of blood glucose (24-h SDBG); 4) Percentage of time in the range of 3.9–10 mmol/L: time in range (TIR); 5) Percentage of time < 3.9 mmol/L or < 3.0 mmol/L: time below range (TBR); 6) Percentage of time > 13.9 mmol/L: time above range (TAR).

### Statistical Analysis

Data were analysed using SPSS software (version 21.0, SPSS, Inc, Chicago, USA). All data were recorded and exported from the CGM 3.0 software analysis system (Medtronic Mini Med, USA). Normally distributed and continuous variables are presented as the mean (standard deviation, SD). Nonnormally distributed variables are presented as medians (interquartile ranges). An independent samples t-test and a rank sum test were used to compare difference between the groups for normally and non-normally distributed data, respectively. P values were two tailed with a significance level of 5%.

## Results

### Baseline Characteristics

The mean age of patients in the basal insulin group was 59.40 ± 11.88 years, and that of the premixed insulin group was 63.14 ± 9.51 years. The percentage of achieving HbA1c <7% in the basal insulin group was 35.1% and that in the premixed insulin group was 34.7%. The clinical and demographic characteristics of both groups were similar, except for the duration of insulin, insulin dose and insulin antibody level, which were all increased in the premixed insulin group (**Table 1**). Patients who were treated with insulin combined with oral agents are shown in **Table 2**. There is a significantly greater frequency of use of insulin secretagogues in the basal insulin group than in the premixed insulin group.

**Table 1:** Baseline characteristics of patients.

Group	Basal Insulin Group (N=199)	Premixed Insulin Group (N=194)	p value
Sex (M/F)	125/74	113/81	0.83
Age (years)	59.40 ± 11.88	63.14 ± 9.51	0.68
HbA1c (%)	7.90 ± 1.69	7.69 ± 1.43	0.33
BMI (kg/m2)	24.92 ± 4.56	24.82 ± 3.10	0.61
Duration of T2DM (years)	15.0 (10.00, 16.50)	13.00 (9.00, 20.00)	0.51
Duration of insulin (years)	5.23 (3.00, 7.71)	6.42 (5.57, 11.00)	<0.01**
Insulin dose (IU/kg/day)	0.30 ± 0.10	0.53 ± 0.17	<0.01**
Fasting C-peptide (ng/ml)	1.20 ± 1.00	1.38 ± 0.99	0.07
Ins-Ab (COI)	4.69 (1.91, 10.02)	10.57 (3.95, 28.07)	<0.01**

Data was shown as mean ± SD or median (first quartile, third quartile). M, male; F, female; HbA1c, glycated haemoglobin; BMI, body mass index; **p < 0.01. Ins-Ab, insulin antibody; COI, OD/CO; OD, absorbance; CO, average absorbance of negative control; The reference range of normal values for antibody is <1 COI.

**Table 2:** Drugs used in addition to insulin.

Group	Basal Insulin Group (N=199)	Premixed Insulin Group (N=194)	p value
Metformin (%)	59.3%	63.9%	0.58
α-glucosidase inhibitor (%)	73.4%	71.1%	0.61
Insulin secretagogues (%)	40.9%	7.8%	<0.01**
DPP-4 inhibitor (%)	9.5%	6.7%	0.48

**p < 0.01.

### The Glucose Profiles

**Figure 2:**
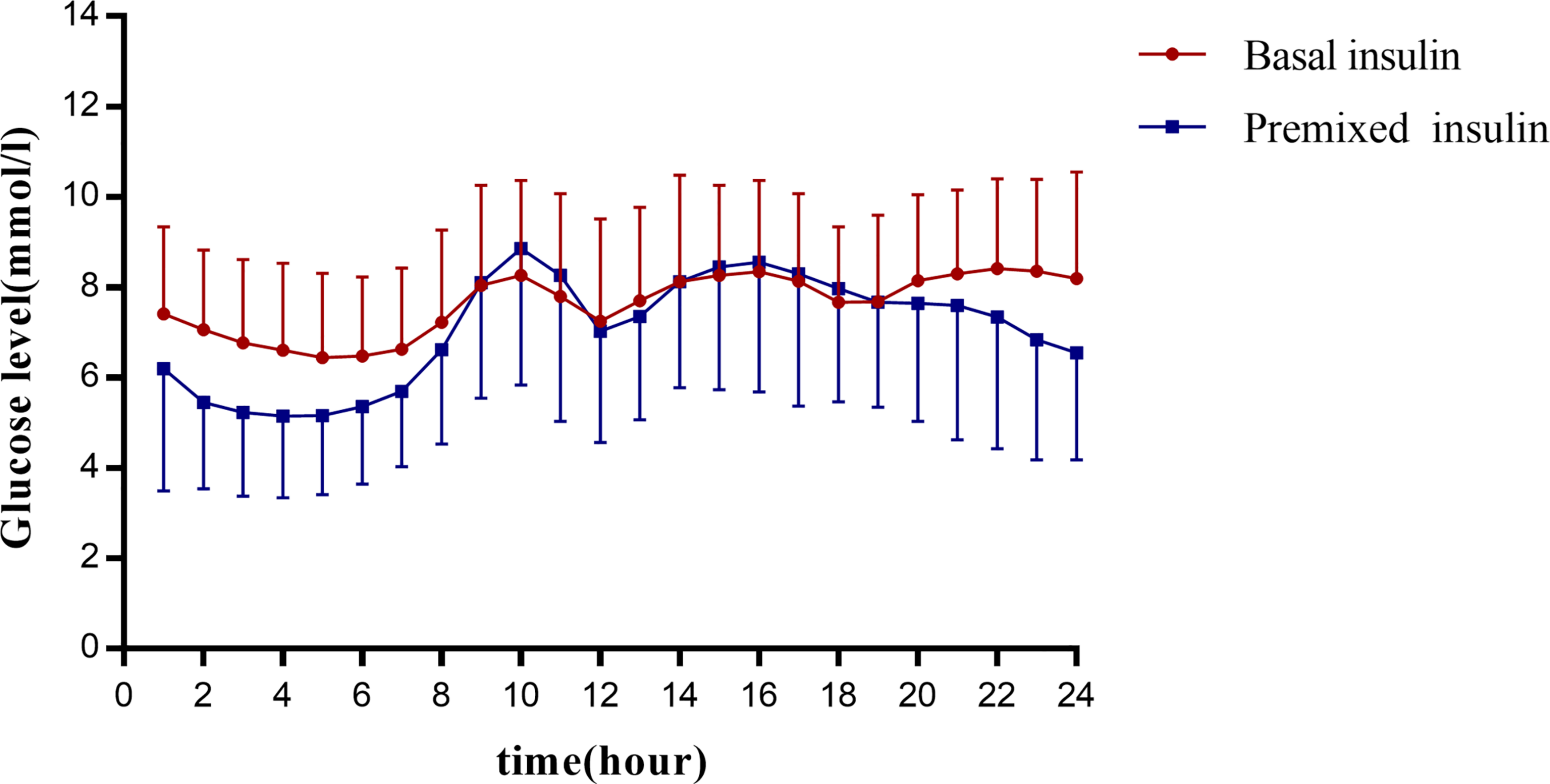
Graph presents the glucose profiles of basal insulin and premixed insulin. The vertical coordinates show the mean glucose ± SD per hour for all patients in each group. The 24 points in each group are connecting to visualize the 24-hour glucose fluctuations in this group of patients. The horizontal coordinates are spaced at 2-hour intervals for each point.

The MBG of the basal insulin group was 9.27 ± 2.38 mmol/L, and that of the premixed insulin group was 8.92 ± 2.74 mmol/L; there was no significant difference between the two groups. The 24-h glucose profiles recorded by CGM during the use of basal insulin and premixed insulin are shown in **Figure 2**.

### Glycaemic Variability

**Figure 3:**
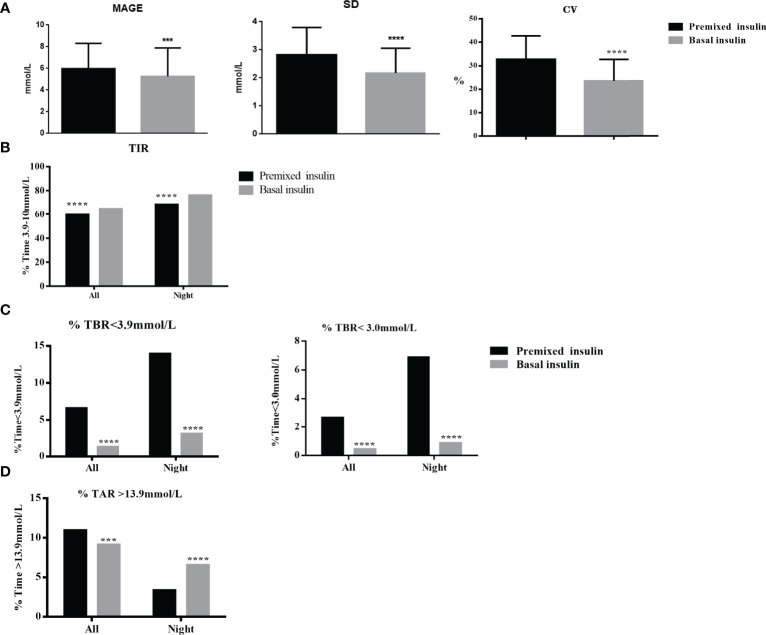
**(A)** GV (MAGE, SD, CV) of the basal insulin group and the premixed insulin group within the full 24-hrs. **(B)** Percentage of TIR (3.0~10 mmol/L) of the basal insulin group and the premixed insulin group within the full 24-hrs and nighttime (00:00–05:59) periods. **(C)** TBR (<3.9 and <3.0 mmol/L) of the basal insulin group and the premixed insulin group within the full 24-hrs and nighttime (00:00–05:59) periods. **(D)** TAR (>13.9 mmol/L) of the basal insulin group and the premixed insulin group within the full 24-hrs and nighttime (00:00–05:59) periods. ***p < 0.001, ****p < 0.0001.

The glycaemic variability, including value of MAGE, SD and CV, was significantly lower in the basal insulin group than these in the premixed insulin group (p<0.01) (**Figure 3A** and **Table 3**).

**Table 3:** CGM index between two groups.

Group		Basal Insulin Group (N=199)	Premixed Insulin Group (N=194)	p value
MBG (mmol/L)		9.27 ± 2.38	8.92 ± 2.74	0.078
GV	MAGE (mmol/L)	4.72 ± 2.53	5.36 ± 2.09	0.035*
	SD (mmol/L)	1.82 ± 0.78	2.54 ± 0.77	<0.01**
	CV (%)	22.87 ± 9.03	36.08 ± 9.77	<0.01**
TIR (%) (3.9-10mmol/L)		64.9 ± 29.3	59.8 ± 23.8	<0.01**
TAR (%) >13.9mmol/L		0.00 (0.00, 0.00)	0.00 (0.00, 5.12)	<0.01**
TBR (%) <3.9mmol/L		0.00 (0.00, 0.35)	9.38 (1.04, 18.06)	<0.01**
TBR (%) <3.0mmol/L		0.00 (0.00, 0.00)	1.74 (0.00, 7.38)	<0.01**

*p < 0.05, **p < 0.01. Data was shown as mean ± SD or median (first quartile, third quartile). MBG, 24-hour mean blood glucose; GV, Glycaemic variability; MAGE, 24-hour mean amplitude of glycaemic excursion; SD, Standard deviation of blood glucose; CV, coefficient of variation; TIR, time in range (3.9-10 mmol/L); TAR, time above target range (>13.9 mmol/L); TBR, time-below-target ranges (<3.9 mmol/L or <3.0 mmol/L).

### Time in Range

The TIR (3.9–10.0 mmol/L) was significantly higher in the basal insulin group compared to the premixed insulin group (64.9% ± 29.3% *vs* 59.8% ± 23.8%, p=0.01) (**Figure 3B** and **Table 3**). The frequency of achieving TIR > 70% had significantly increased in the basal insulin group compared to the premixed insulin group (51.8% *vs* 40.2%, p<0.01).

### Time Below Range

There were no severe hypoglycaemic events reported or dose adjustment issues occurred during the whole study. TBR < 3.9 mmol/L and < 3.0 mmol/L were significantly lower in the basal insulin participants compared to the premixed insulin participants, especially at night (0:00–05:59 h) (p<0.01) (**Figure 3C** and **Table 3**).

### Time Above Range

TAR>13.9 mmol/L was significantly higher in the premixed insulin group (3.82 (0.00, 16.15) *vs* 0.00 (0.00, 15.97), p=0.01) compared to the basal insulin group (**Figure 3D** and **Table 3**).

### Intergroup Clinical Characteristics of Patients With Different HbA1c Values

For the subgroups with HbA1c <7%, the value of MAGE was decreased in basal insulin group compared to premixed insulin group (4.72 ± 2.53 and *vs* 5.36 ± 2.09, p<0.01). TBR<3.9mmol/L in basal insulin group was lower compared to premixed insulin group (0.00 (0.00,0.35) *vs* 9.38 (1.04, 18.06), p < 0.01). For the subgroups with 7% ≤ HbA1c ≤ 9%, the GV and TBR of the premixed insulin group showed a significant increase than those in the basal insulin group (p<0.05), as well as the insulin antibody level. For the subgroups with HbA1c > 9%, there was no significant difference in CGM parameters between the two groups (**Table 4**).

**Table 4:** Clinical characteristics of patients with different HbA1c levels.

Group	HbA1c<7%			7%≤HbA1c≤9%			HbA1c>9%		
	Basal Insulin (N=69)	Premixed Insulin (N=68)	P value	Basal Insulin (N=80)	Premixed Insulin (N=93)	P value	Basal Insulin (N=50)	Premixed Insulin (N=33)	P value
MBG (mmol/L)	7.54±1.57	7.15±1.51	<0.01**	9.62±2.40	9.28±2.21	0.412	10.53±2.43	11.58±3.50	0.211
MAGE (mmol/L)	4.72±2.53	5.36±2.09	0.035*	5.43±2.48	6.33±2.13	0.011*	5.78±2.88	6.28±3.10	0.389
SD (mmol/L)	1.82±0.78	2.54±0.77	<0.01**	2.28±0.84	3.04±0.95	<0.01**	2.46±0.98	2.85±1.22	0.182
CV (%)	22.87±9.03	36.08±9.77	<0.01**	24.25±9.61	33.35±8.70	<0.01**	23.43±8.59	24.62±9.18	0.612
TIR (%)	86.46 (72.05, 96.35)	73.96 (61.28, 80.90)	<0.01**	64.76 (36.63, 86.72	60.76 (43.58, 75.87)	0.287	48.78 (19.53, 74.83)	37.84 (19.44, 59.20)	0.235
TBR <3.9 mmol/L (%)	0.00 (0.00, 0.35)	9.38 (1.04, 18.06)	<0.01**	0.00 (0.00, 0.00)	1.04 (0.00, 7.29)	<0.01**	0.00 (0.00, 0.00)	0.00 (0.00, 0.00)	0.508
TBR <3.0 mmol/L (%)	0.00 (0.00, 0.00)	1.74 (0.00, 7.38)	<0.01**	0.00 (0.00, 0.00)	0.00 (0.00, 2.43)	<0.01**	0.00 (0.00, 0.00)	0.00 (0.00, 0.00)	0.321
C-Peptide (ng/ml)	1.22±0.83	1.32±0.73	0.53	1.36±0.38	1.47±1.13	0.58	1.05±1.82	1.32±1.22	0.27
Ins-Ab (COI)	3.10 (1.13, 11.06)	9.25 (2.89, 29.74)	<0.01**	5.06 (2.37, 10.09)	13.43 (5.53, 30.31)	<0.01**	4.31 (1.98, 9.14)	8.42 (2.68, 13.98)	0.13

Data was shown as mean ± SD or median (first quartile, third quartile). *p < 0.05, **p < 0.01. HbA1c, glycated haemoglobin; MBG, 24-hour mean blood glucose; GV, Glycaemic variability; MAGE, 24-hour mean amplitude of glycaemic excursion; SD, Standard deviation of blood glucose; CV, coefficient of variation; TIR, time in range (3.9-10 mmol/L); TAR, time above target range (>13.9 mmol/L); TBR, time-below-target ranges (<3.9 mmol/L or <3.0 mmol/L); Ins-Ab, insulin antibody; COI, OD/CO; OD, absorbance; CO, average absorbance of negative control; The reference range of normal values for antibody is <1 COI.

### Intragroup Clinical Characteristics of Patients With Different HbA1c Levels

In the basal insulin group and premixed insulin group, there was no significant difference in clinical characteristics between patients with HbA1c < 7% and patients with HbA1c ≥ 7% (**Tables 5**, **6**).

**Table 5:** Clinical characteristics of patients with subdivided basal insulin group.

Basal Insulin	HbA1c<7% (N=69)	HbA1c≥7% (N=130)	p value
Age (years)	59.64 ± 10.22	59.28 ± 12.71	0.83
Fasting C-peptide (ng/ml)	1.20 ± 0.08	1.20 ± 0.10	0.98
Ins-Ab (COI)	7.7 ± 0.99	9.12 ± 1.32	0.39
Duration of T2DM (years)	14.24 ± 6.02	13.21 ± 6.13	0.25
Duration of insulin (years)	5.76 ± 3.12	5.20 ± 3.18	0.22
Insulin dose (IU/kg/day)	0.28 ± 0.08	0.32 ± 0.13	0.12

Data was shown as mean ± SD. HbA1c, glycated haemoglobin; Ins-Ab, insulin antibody; COI, OD/CO; OD, absorbance; CO, average absorbance of negative control; The reference range of normal values for antibody is <1 COI.

**Table 6:** Clinical characteristics of patients with subdivided premixed insulin group.

Premixed Insulin	HbA1c<7% (N=68)	HbA1c≥7% (N=126)	p value
Age (years)	63.09 ± 9.32	63.15 ± 9.09	0.86
Fasting C-peptide (ng/ml)	1.32 ± 0.09	1.43 ± 0.10	0.48
Ins-Ab (COI)	17.01 ± 2.27	18.36 ± 1.73	0.64
Duration of T2DM (years)	12.75 ± 6.92	14.07 ± 6.82	0.20
Duration of insulin (years)	7.91 ± 6.78	7.59 ± 5.95	0.73
Insulin dose (IU/kg/day)	0.49 ± 0.02	0.47 ± 0.02	0.63

Data was shown as mean ± SD. HbA1c, glycated haemoglobin; Ins-Ab, insulin antibody; COI, OD/CO; OD, absorbance; CO, average absorbance of negative control; The reference range of normal values for antibody is <1 COI.

## Discussion

In this cross-sectional study, our results showed although there was not a gap of actual HbA1c between the two groups, TIR in the basal insulin group was greater than that in the premixed insulin group. Although the MBG of both groups were similar, the basal insulin group had lower GV, overall hypoglycaemia and nocturnal hypoglycaemia than the premixed insulin group.

In the present study we found that the basal insulin group had similar levels of C-peptide as the premixed insulin group. C-peptide is produced with an equal amount of insulin and is the best measure of endogenous insulin secretion in patients with diabetes (15). The key current clinical role of C-peptide is to assist classification and management of insulin-treated patients. C-peptide is inversely associated with glycaemic variability and post-meal glucose rise in both Type 1 and Type 2 diabetes (16) and is inversely associated with response to prandial insulin in experimental conditions in a mixed population with diabetes. Given that low C-peptide is associated with higher glucose variability, the absence of statistically significant difference in C-peptide between basal insulin and premixed insulin groups suggests that residual beta-cell function was not responsible for the CGM differences observed in this study.

Severe hypoglycaemia causes patients to show signs of an insufficient energy supply to the central nervous system, such as drowsiness, disturbance of consciousness, nonsense, and even coma and death (17). It was reported that 80% of diabetes specialists feel they are unable to proactively treat the disease because of the risk of hypoglycaemia (18); thus, treatment that does not result in hypoglycaemia is very important for diabetic patients. Basal insulin treatment has a lower risk of hypoglycaemia, which enables more aggressive treatment and is easier to use with less variation (19). In this study, symptomatic hypoglycaemia and severe hypoglycaemia did not occur, but the TBR < 3.9 mmol/L and TBR < 3.0 mmol/L were significantly higher in the premixed insulin group than in the basal insulin group. More importantly, nocturnal hypoglycaemia also causes severe damage to patients if it recurs and is undetectable, and even mild asymptomatic episodes can lead to further impairment and defective counterregulatory responses to subsequent events (20). In addition to an increased risk for future episodes, the effects of nocturnal hypoglycaemia that occur during the day, such as fatigue, impaired mood and higher calorie intake and weight gain, considerably lower quality of life (21). Our study found the TBR < 3.9 mmol/L and TBR < 3.0 mmol/L in the premixed insulin group at night were both higher than those in the basal insulin group.

In addition, in this study, we divided patients according to the level of HbA1c across the two groups. For the subgroups with HbA1c <7%, the GV, TBR < 3.9 mmol/L and TBR < 3.0 mmol/L of the premixed insulin group were higher than those in the basal insulin group. Patients in the premixed insulin group do not have lower C-peptide levels compared to the basal insulin group and have elevated insulin antibody (IA), as well as a higher incidence of hypoglycaemia. Longer duration of insulin use which made possible exposure to more types of insulin are potential causes for significantly greater insulin antibody levels in the premixed insulin group. Administration of exogenous animal insulin for the treatment of diabetes often induces the production of IA (22). In recent years, the usage of recombinant human insulin preparations and human insulin analogues has significantly reduced but not entirely suppressed the incidence of IA development. These antibodies might affect a patient’s glycaemic control due to their tendency to bind and/or release insulin in an unpredictable fashion. The higher circulating IA was associated with increased MAGE in T2DM patients (23), thus greater GV may be due to greater IA levels in the premixed insulin group potentially, which indicate that those patients with elevated IA levels should receive GV assessment and individualized treatment. For the subgroups with HbA1c <7% and 7% ≤ HbA1c ≤ 9%, patients who use premixed insulin have a higher IA level, the TIR was not higher than patients in the basal insulin group, however the incidence of hypoglycaemia and GV were both higher in premixed insulin group. For the subgroups with HbA1c > 9%, the MBG and TIR of patients in both groups did not meet the standard, and the C-peptide levels of patients who use basal insulin were not high.

C-peptide measurement is critical in insulin selection for it can reflect islet function of patients, the regimen for patients who use premixed insulin could be considered to use basal insulin if they present higher incidence of hypoglycaemia, greater GV and better islet function. CGM is an important device on detection of asymptomatic hypoglycaemia and hypoglycaemia, and should be fully considered when choosing an insulin regimen. Although the lower price of premixed insulin is one of the reasons for Chinese patients and physicians tend to use premixed insulin. It is still important not to be price oriented when making the choice of insulin type, as the above mentioned, islet function, frequency of hypoglycaemia, and GV are all factors to be considered.

Limitations should also be addressed. Firstly, findings using a larger sample size may be more convincing. Secondly, the differential use of insulin secretagogues, insulin dosing, and duration of insulin use between the two groups could partly contribute to result bias. Thirdly, the cross-sectional study design is insufficiently to answer persistent effects on the glycaemic control process of the same individual with premixed insulin or basal insulin therapy. Finally, our study was limited to a single centre, which should be expanded. We hope to follow up these subjects and expand the sample in the future.

## Conclusions

In summary, compared with basal insulin, the patients who currently use premixed insulin had more severe GV, a smaller TIR and a higher incidence of hypoglycaemia. Among people who use premixed insulin and have an HbA1c < 7%, more attention needs to be paid on hypoglycaemic events and asymptomatic hypoglycaemia. If necessary, the insulin regimen should be adjusted.

## Data Availability Statement

The raw data supporting the conclusions of this article will be made available by the authors, without undue reservation.

## Ethics Statement

The studies involving human participants were reviewed and approved by Ethics Committee of Nanjing First Hospital. The patients/participants provided their written informed consent to participate in this study.

## Author Contributions

HW and YZ analyzed data and wrote the manuscript. YW, TC, and YH organized data. TJ, BD, and XS modified the manuscript. HL and JM conceived, and directed the study. All authors contributed to the article and approved the submitted version.

## Funding

This study was partly supported by the National Key R&D Program of China (No. 2018YFC1314103), the Xinghuo Talent Program of Nanjing First Hospital (To YZ), and Jiangsu Innovative and Entrepreneurial Talent Programme (No.JSSCBS20211546).

## Conflict of Interest

The authors declare that the research was conducted in the absence of any commercial or financial relationships that could be construed as a potential conflict of interest.

## Publisher’s Note

All claims expressed in this article are solely those of the authors and do not necessarily represent those of their affiliated organizations, or those of the publisher, the editors and the reviewers. Any product that may be evaluated in this article, or claim that may be made by its manufacturer, is not guaranteed or endorsed by the publisher.
